# Reliability and Validity of Plantar Pressures and the Modified ICPBL Test vs. Telemetry for Diagnosing Anatomical Discrepancies: A Pilot Study

**DOI:** 10.3390/life16040612

**Published:** 2026-04-07

**Authors:** Arian Marcelino Argemi, Dan Iulian Alexe, Ismael Ortuño Soriano, Ignacio Zaragoza García, Alvaro Saura Sempere, Rebeca Bueno Fermoso, Álvaro Gómez Carrión, Rubén Sánchez-Gómez

**Affiliations:** 1 Nursing Department, Faculty of Nursing, Physiotherapy and Podiatry, Universidad Complutense de Madrid, 28040 Madrid, Spain; a.marcelino.argemi@gmail.com (A.M.A.); iortunos@ucm.es (I.O.S.); izaragoz@ucm.es (I.Z.G.); alvarosaura@gmail.com (A.S.S.); rebueno@ucm.es (R.B.F.); alvaroalcore@hotmail.com (Á.G.C.); 2 Department of Physical and Occupational Therapy, Faculty of Movement, Sports and Health, Sciences, “Vasile Alecsandri” University of Bacău, 600115 Bacău, Romania; alexedaniulian@ub.ro; 3 Instituto de Investigación Sanitaria Hospital Clínico San Carlos (IdISSC), 28040 Madrid, Spain; 4 Instituto de Investigación Sanitaria imas12, Grupo Invecuid, 28041 Madrid, Spain

**Keywords:** anatomic discrepancies, plantar pressures, telemetry, Modified ICPBL test

## Abstract

**Background/Objectives:** Several methods have been proposed to assess lower limb-length discrepancies; however, none have demonstrated sufficient reliability and validity for diagnosing anatomical discrepancies (DA). Objectives: This study primarily aims to evaluate the accuracy of two traditional tests—the Modified Iliac Crests Palpation and Pelvimeter with Blocks test (ICPBL) and plantar pressure analysis—by comparing them with the gold standard telemetry (TE) method for diagnosing DA. The secondary objective is to assess the intra-rater reliability of these two tests and determine their potential applicability in clinical settings. **Methods:** Thirty subjects between the ages of 20 and 80 were enrolled in the present prospective, cross-sectional diagnostic accuracy pilot study; thirteen with a positive TE for DA of more than 3 mm were classified into the group with the condition DA, and 17 were classified into the group without the condition DA. Pelvic tilting and plantar pressures were evaluated. **Results:** The TE revealed a difference of 8.09 ± 3.24 mm between the short and long limbs, while subjects without DA had only a 0.41 mm difference (*p* < 0.001). Similarly, the Modified ICPBL test showed a 4.38 ± 2.10 mm difference in subjects with DA, compared to 0.51 ± 0.53 mm in those without DA (*p* < 0.001). Additionally, plantar pressure measurements supported these findings, with a difference of 5.17 ± 3.28 kg/cm^2^ between the short and long limbs in subjects with DA, versus 2.28 ± 1.77 kg/cm^2^ in subjects without DA (*p* < 0.05). The area under the receiver operating characteristic (ROC) curve was 0.783 (95% CI: 0.456–0.877) for plantar pressures and 1.000 (95% CI: 0.742–0.942) for the Modified ICPBL test. **Conclusions:** The Modified ICPBL and plantar pressure tests demonstrated high diagnostic accuracy within the sample studied, suggesting they are useful tools for supporting the diagnosis of DA. In this pilot study, the Modified ICPBL showed very high discriminative ability, while plantar pressure testing demonstrated moderate sensitivity. Both methods may serve as preliminary practical alternatives to telemetry TE, potentially reducing X-ray exposure; however, these results should be interpreted with caution due to the limited sample size and the specific clinical setting of this study.

## 1. Introduction

The clinical assessment of lower limb length discrepancies encompasses a wide array of diagnostic methodologies [1,2,3,4,5,6,7,8,9,10], often complicated by heterogeneous terminology and numerous intervening anatomical variables [11,12,13,14,15]. Traditionally, these conditions are bifurcated into anatomical discrepancy (DA)—defined as a structural, measurable difference in the osseous length of the femur or tibia [2]—and functional discrepancy, which arises from soft tissue imbalances, pelvic torsions, or joint misalignments that alter the perceived limb length [2,6]. A discrepancy is formally classified as DA when it exceeds a 3 mm threshold [7], a benchmark typically validated through load-bearing anteroposterior telemetry (TE). Although TE remains the diagnostic gold standard due to its high precision [10,12], the necessity for specialized equipment and the associated exposure to ionizing radiation [14,15,16] have driven the search for more accessible, non-invasive screening alternatives.

The etiology of DA is diverse, ranging from congenital malformations to acquired pathologies such as growth plate disturbances, osteonecrosis, or post-traumatic fractures [8,9]. Conversely, functional discrepancy is frequently linked to compensatory biomechanical adaptations, including foot pathomechanics and gait alterations [8,9,10,11,12]. While some research suggests that DA acts as a primary catalyst for functional impairments [10], the clinical significance of mild asymmetries, particularly their role in triggering low back pain, remains a subject of ongoing debate [11]. Despite these complexities, manual clinical tests [12] remain a staple in practice, although they often demonstrate lower reliability compared to radiographic imaging [7,13].

### 1.1. Palpation of the Iliac Crests and Pelvimeter with Blocks (ICPBL)

Among non-radiological assessments, the evaluation of iliac crest height is one of the most widely utilized techniques. However, the ICPBL method is frequently compromised by significant intra- and inter-examiner bias [17]. Factors such as difficulty in accurately palpating bony landmarks and the presence of pelvic rotations can lead to substantial measurement errors when compared to TE [2,18,19]. Recent investigations have yielded conflicting data; while some studies report poor correlation and only moderate validity for this manual approach [4], a significant systematic review [20] recently identified it as one of the most reliable manual methods for assessing DA when compared against other clinical tools such as tape measures or visual assessments.

### 1.2. Plantar Pressure Analysis

Podobarometry has emerged as a valuable tool for detecting biomechanical abnormalities and postural shifts associated with limb asymmetry [17,21]. In cases of functional restrictions, plantar pressure platforms have proven effective in quantifying alterations in load distribution [21]. Previous research using simulated discrepancies (utilizing blocks in healthy subjects) reported a high intraclass correlation index [22], generally observing a significant increase in load under the shorter limb [23,24]. However, a critical gap exists in the current literature: most evidence is based on artificial simulations rather than true DA cases, and there is a notable lack of standardized protocols or clear diagnostic definitions across existing studies [17,18,19,20,21,22,23,24].

The theoretical rationale for comparing ICPBL and podobarometry lies in their different approaches to capturing the kinetic chain’s response to asymmetry. While pelvic leveling (ICPBL) measures the proximal structural compensation—directly quantifying the frontal plane tilt of the pelvis caused by an unequal osseous foundation—plantar pressure analysis captures the distal kinetic adaptation [4,15]. Theoretically, a DA shifts the body’s center of mass toward the shorter limb to maintain postural equilibrium, increasing the vertical ground reaction forces and, consequently, the peak pressures under that foot [7,13,15]. Comparing these two methods allows us to determine whether structural pelvic misalignment or dynamic load redistribution is a more sensitive indicator for early-stage DA screening.

### 1.3. Rationale, Objectives, and Hypotheses

Despite the clinical use of ICPBL and plantar pressure analysis, comparative research against the TE remains scarce. In this study, a 3 mm diagnostic threshold was established based on the high precision of digital telemetry [3]. While traditional literature focuses on discrepancies >5 mm [2], recent evidence suggests that asymmetries of 3 mm can significantly alter postural control [7,8]. Thus, this study evaluates the technical detection sensitivity of these tools to distinguish early diagnostic capability from broader clinical symptom onset.

Our primary objective is to determine which non-invasive method provides a more valid screening for DA and to establish specific cut-off values. We hypothesize that the ICPBL method will demonstrate superior validity and reliability compared to plantar pressure measurements. Furthermore, we anticipate that our Modified ICPBL test will detect DA more sensitively at lower thresholds, providing a precise screening framework to minimize unnecessary radiographic radiation exposure.

## 2. Materials and Methods

This study was reviewed and approved by the Ethics Committee of the San Carlos Hospital, Madrid, Spain, with number C.I. 22/740-E. Data collection was conducted at the Pododinámica clinic (Madrid, Spain) between January 2023 and June 2023 [6 months], The study was conducted in accordance with the ethical principles outlined in the Declaration of Helsinki. All participants read and signed the informed consent.

### 2.1. Study Design and Sample Size

In this prospective, cross-sectional diagnostic accuracy study with grouping based on the reference standard, a convenience sampling technique was employed. Participants were identified through clinical screening at the private Pododinamica^®^ clinic and via the public recruitment of volunteers with suspected limb length discrepancy. While STROBE guidelines were followed to ensure reporting quality, we acknowledge that the recruitment was not randomized.

Regarding sample size, the study was designed to ensure sufficient power for both comparative and diagnostic accuracy analyses. Based on previous research [24], it was estimated that to detect significant differences in anatomical features between groups with a statistical power of 80%, a 95% confidence interval (α = 0.05), and a moderate effect size (Cohen’s d = 0.5), at least 12 subjects per group were required. Furthermore, a formal power analysis for diagnostic accuracy was conducted based on the expected Area Under the ROC Curve [25]. Assuming a null hypothesis of area under curve = 0.5 and an alternative hypothesis of area under curve = 0.80, a total sample of 30 participants (ratio 1.3:1) provides a statistical power of 84.1% (α = 0.05) to detect significant discriminative ability. According to Buderer’s formula, this sample size also allows for the estimation of sensitivity and specificity with a pre-specified precision, confirming that the total of 30 participants (13 with DA and 17 without DA) is statistically adequate for this pilot diagnostic phase.

The inclusion criteria were as follows: healthy male and female subjects (ages 20–80) with a confirmed anatomical DA of ≥3 mm [3], as measured by the gold-standard telemetry (TE) under weight-bearing conditions. This threshold was selected because, with high-precision imaging and standardized positioning, a 3 mm difference exceeds the absolute measurement error typically reported for these modalities (≈2.6–3.6 mm) [7,26,27]. This threshold reflects the limit of detection for reliable imaging rather than a clinically relevant difference, as biomechanical or symptomatic consequences are generally observed with discrepancies ≥5–10 mm [28]; no clinical history of lower limb trauma; normal joint range of motion and axial alignment of the lower limbs [29]; neutral foot posture index. Exclusion Criteria: subjects with balance issues, uncorrected hearing or vision problems; subjects diagnosed with hyper lordosis [30]; subjects currently undergoing treatment with any medication affecting the nervous system.

### 2.2. Procedure and Technical Considerations

The TE were performed with the radiology equipment from General Electric, model DISCOVERY^®^ 656 HD//JEDI 80RD 1T, with serial number MSS1900001//211137W65. The X-ray tube model is MX 100 0.6 1.25 12.5°, with the serial number 136714B13 (GE HealthCare, Chicago, IL, USA). The generator has the following technical specifications: maximum voltage of 150 kV, maximum current of 1000 mA. The distance from the device to the subject was 180 cm, with a height of 20–25 cm from the feet to the floor. The exposure was adjusted with doses based on each patient.

To assess and detect DA in TE, the AutoCAD^®^ software 2024 version was used (Autodesk Inc., San Rafael, CA, USA) [31].

#### 2.2.1. Instrumentation and Calibration Protocols

To ensure high measurement accuracy, the digital pelvimeter was calibrated before each session using a high-precision digital level (0.05° resolution) to verify the horizontal zero-point. The Footchecker^®^ platform features a registration surface of 400 × 400 mm with 2704 calibrated resistive sensors and a sampling frequency of 100 Hz. The device operates within a pressure range of 0.10 kg/cm^2^ a 12.24 kg/cm^2^ with a hysteresis of less than 3%.

Before the study, the platform underwent standard factory calibration and pre-session zero-loading verification. The reliability of this system has been previously validated, demonstrating high internal consistency (ICC > 0.90) for static pressure distribution [28,32]. All sessions were conducted in a controlled clinical environment at a constant temperature (approx. 22 °C) to prevent thermal interference with the sensors. To minimize systematic bias, the order of the Modified ICPBL and plantar pressure assessments was randomized for each participant, and a 5 min acclimatization period was provided before data collection.

#### 2.2.2. Modified ICPBL Test

The original ICPBL method involved palpating the iliac crests to estimate height discrepancy, corrected with lifts until pelvic balance was perceived [11,33,34,35]. In our Modified ICPBL method, 1 mm rigid blocks and a digital pelvimeter were used to enhance detection accuracy. Subjects were instructed to perform a brief “soldier’s march” in place until finding a comfortable stance, maintaining an upright, relaxed posture with knees fully extended and eyes facing forward [34].

The examiner, positioned behind the subject with arms extended, palpated the iliac crests [35] and placed the mobile arms of the digital pelvimeter over them. Pelvic tilt was corrected by placing 1 mm blocks under the shorter limb until equilibrium was achieved. The total height of the blocks was summed to estimate the limb-length difference. Each measurement was repeated three times to ensure consistency. Despite these refinements, the method remains limited by potential palpation difficulties and individual postural variations.

#### 2.2.3. Plantar Pressure Assessments

Static plantar pressure analysis was performed using the Footchecker^®^ platform and IST Footchecker^®^ software (v. 2.9.9.0) to record pressure in kilopascals (kPa) and support surface in square centimeters (cm^2^) [30,32]. To achieve a natural position, patients performed 5 steps of a “soldier’s march” before holding a static position.

A 30 s stabilization window was implemented before recording the mean of three 10 s captures. This protocol was designed to filter out transient postural sway and ensure that the recorded values reflected a true steady-state distribution. Between each trial, the software was restarted to allow for platform recalibration. Data collected included total support area and the percentage of total and partial pressure (% forefoot and % hindfoot) for each foot.

### 2.3. Statistical Analysis

The normality of the sample was tested using the Kolmogorov–Smirnov test with Lilliefors correction, considering a normal distribution if *p* > 0.05. For each continuous variable, we calculated the mean ± standard deviation (±SD) and a 95% confidence interval (CI). To determine the reliability of the measurements, the intraclass correlation coefficient (ICC) was calculated. Specifically, a two-way mixed-effects model with absolute agreement and single-measures (ICC 3,1) was employed to assess intra-examiner reliability. ICC values range from 0 to 1, with higher values indicating greater reliability. Following widely accepted guidelines by Koo and Li [36], ICC values were interpreted as follows: values below 0.5 indicate poor reliability, between 0.5 and 0.75 indicate moderate reliability, between 0.75 and 0.9 indicate good reliability, and values above 0.9 indicate excellent reliability. These thresholds were applied to interpret the reliability of the measurement systems and intra-examiner assessments in the present study. In addition, Bland–Altman analysis was used to assess the agreement between two measurement methods. This method evaluates the mean difference and the limits of agreement, providing a quantitative and graphical assessment of measurement consistency. It is widely applied to determine whether different methods can be used interchangeably. To assess differences in the dependent variables between the with or without DA condition groups, either the unpaired Student’s *t*-test or the Mann–Whitney U test was applied, depending on whether the sample data met the assumptions of normality. To determine the sensitivities and specificities of the tests and establish optimal diagnostic cutoff values for diagnosing DA, ROC curves [37,38] were plotted. This approach was chosen because ROC analysis allows the assessment of sensitivity and specificity across all thresholds and provides the area under the curve as a measure of overall discriminative ability. Cutoff points were selected using the Youden index, which maximizes the sum of sensitivity and specificity to balance false positives and false negatives. The precision of the diagnostic accuracy parameters (sensitivity, specificity, and area under curve) was assessed using 95% Confidence Intervals (95% CI) calculated via the Clopper–Pearson exact method. A 95% confidence interval was calculated for the area under the curve and its corresponding limits to evaluate the precision of the estimates and compare the ROC area against the null hypothesis value of 0.5. For all analyses, *p*-values < 0.05 (within a 95% CI) were considered statistically significant. Data analysis was performed using SPSS software, version 19.0 (SPSS Science, Chicago, IL, USA).

## 3. Results

**Figure 1:**
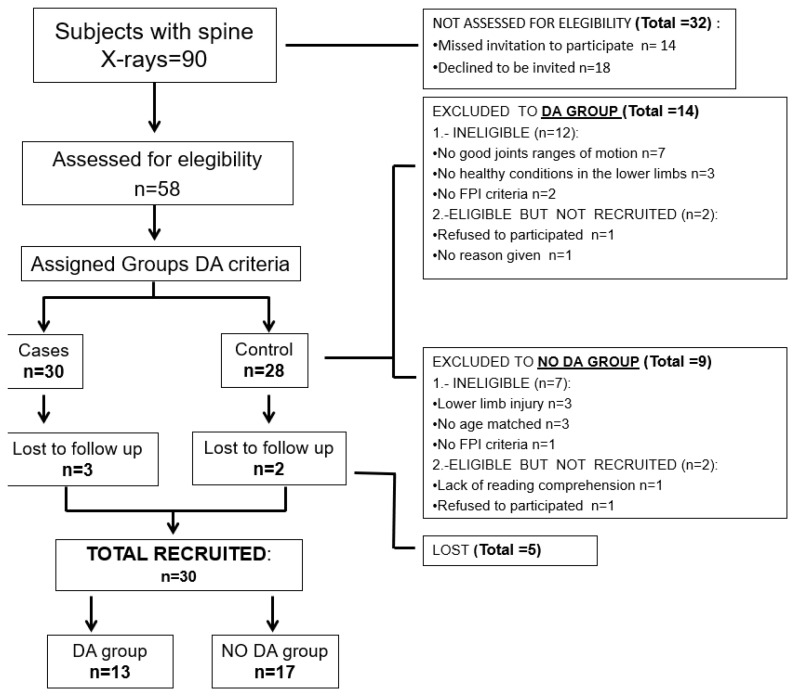
Participant flow chart-recruitment.

Fifty-eight subjects agreed to participate in the study. No missing data were recorded for the index tests or reference standard. After establishing a 3 mm threshold, 30 subjects were selected: 13 were classified into the DA condition group and 17 to the without DA condition group. The remaining 28 subjects were excluded solely for orthopedic, anthropophysical, or social reasons (Figure 1). The data were not normally distributed (*p* < 0.05). The sociodemographic data and their relationship between the exploratory tests and the DA and non-DA groups are shown in Table 1.

Statistically significant differences were observed between the DA and non-DA groups across all primary study variables (Table 1). Both TE and the Modified ICPBL test showed highly significant discrepancies in the DA group compared to the control group (*p* < 0.001). These findings were further supported by plantar pressure analysis, which also revealed significantly higher pressure imbalances in subjects with anatomical discrepancies (*p* = 0.024).

**Figure 2:**
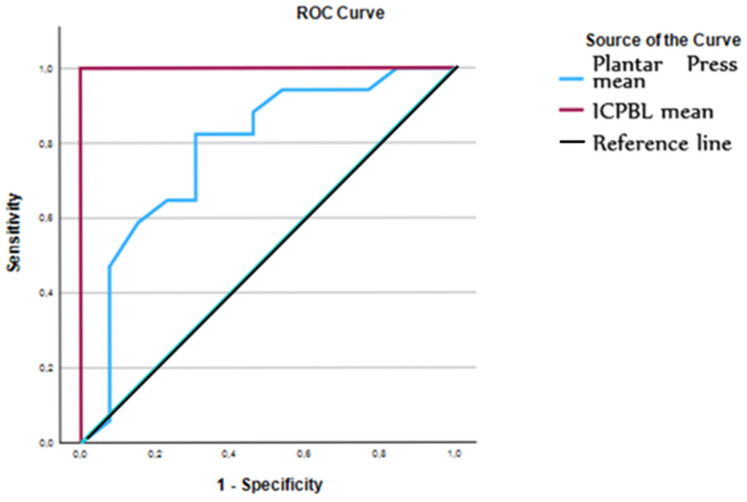
Area under the curve ROC for plantar pressures test (blue line with curve and peaks) and Modified ICPBL test (purple 90° line)—receiver operating characteristics, with good and acceptable distribution. Area Under the Curve; 95% CI: 95% Confidence Interval. CI for sensitivity and specificity were calculated using the Clopper–Pearson exact method. Post hoc power analysis for AUC yielded 84.1% (α = 0.05).

The ROC curve analysis revealed significant diagnostic accuracy for both methods (Figure 2 and Table 2). The plantar pressure test showed an area under the curve of 0.783, with a sensitivity of 69.2% and a specificity of 82.4% at a cutoff of 2.83 kg/cm^2^. In comparison, the Modified ICPBL test demonstrated perfect discriminative ability in this pilot sample, with an area under the curve of 1.000 and 100% sensitivity and specificity at a cutoff of 1.88 mm (Table 3). These results indicate that the Modified ICPBL provides a superior predictive value for identifying anatomical discrepancies compared to plantar pressure analysis.

**Figure 3:**
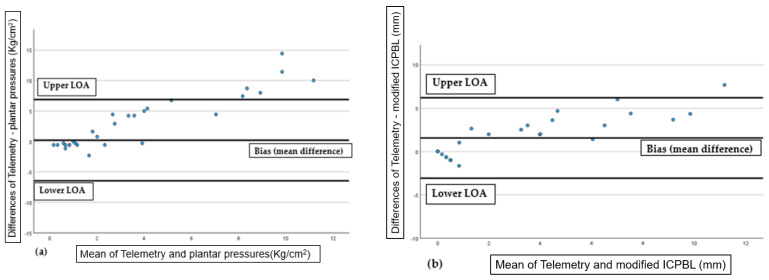
**Bland–Altman plots showing agreement between methods.** (**a**) Gold standard (Telemetry) vs. Plantar pressures; (**b**) Gold standard (Telemetry) vs. Modified ICPBL. The *x*-axis represents the mean of the two measurements, while the *y*-axis shows the difference between them. The solid central line represents the mean difference (bias), and the dashed lines represent the 95% Limits of Agreement (LoA), calculated as bias ± 1.96 SD. These limits define the interval within which 95% of the differences between the gold standard and the alternative method are expected to fall, providing a measure of clinical interchangeability.

For Figure 3a (DA vs. plantar pressures), the mean difference or bias was 0.20, with 95% limits of agreement (LoA) ranging from lower LoA −6.45 to upper LoA 6.87 (±3.40 SD). For Figure 3b (DA vs. Modified ICPBL), the mean difference was 1.54, and the limits of agreement (LoA) extended from Lower LoA −3.09 to Upper LoA 6.19 (±2.37 SD). These values summarize the bias and variability in agreement between each alternative method and the gold standard. These results and the values distribution of the graphic demonstrate good overall agreement with acceptable bias, supporting the reliability of both alternative methods compared to the gold standard DA. The limits of agreement indicate consistent performance across the measurement range, suggesting these methods are viable substitutes in relevant clinical contexts.

Table 4 presents the positive predictive values (PPV) and negative predictive values (NPV) for plantar pressures and the Modified ICPBL test at various DA prevalence rates. At a 35.5% DA prevalence [22] the PPV for plantar pressures is 67.9%, indicating that approximately two-thirds of positive results are true positives. As prevalence increases to 50% and 80% [22], the PPV rises to 79.7% and 94.0%, respectively, reflecting improved test accuracy in higher-prevalence populations. Conversely, the NPV decreases from 82.9% at 35.5% prevalence to 40.1% at 80% prevalence, suggesting that negative results are less reliable in high-prevalence groups. In contrast, the Modified ICPBL test demonstrates perfect discrimination with 100% PPV and NPV across all prevalence levels, consistent with its reported sensitivity and specificity of 100%.

## 4. Discussion

The main goal of the present study was to determine which manual test was the most accurate for properly diagnosing DA, according to the gold standard TE. In addition, we wanted to set the preliminary cut-off values of sensitivity and specificity for these tests.

### 4.1. Modified ICPBL Test

The results obtained with the Modified ICPBL test achieved an apparent 100% accuracy for a difference of 1.88 mm between the long and short sides. While this suggests high diagnostic potential within our sample, it must be explicitly stated that such perfect diagnostic discrimination is unusual in clinical research and should be interpreted with extreme caution. This performance likely reflects the limited sample size and potential sampling bias inherent in a pilot study, rather than an absolute rule for the general population. As noted in high-impact methodological reviews, when the complexity of the analysis—including ROC curves and Bland–Altman plots—is high relative to the number of events (in this case, positive cases of DA), the model may capture random noise rather than a true biological signal, a phenomenon known as overfitting [36,37,38,39]. Although the point estimates for the Modified ICPBL test suggest perfect diagnostic accuracy, the reported 95% CI reflects the statistical uncertainty associated with our pilot sample size [39]. These intervals indicate that while the test is highly promising, its performance in a broader population might vary.

Our findings contrast with previous studies using similar methods, such as the pelvimeter or the Blocks Method, which reported lower reliability or no relationship with TE [4,19,40]. Clarke et al. [1] reported proper diagnosis in only 16 of 60 subjects when discrepancies were <5 mm, compared to our cut-off of 1.88 mm. Similarly, Gómez-Aguilar et al. [4] reported an intra-examiner error of approximately 1.5 mm and a moderate Kappa index for agreement with TE. These discrepancies emphasize the challenges of manual tests, where the rigor of the technique, derived from the examiner’s experience, is crucial [41]. In our study, the examiner’s 15 years of clinical practice likely contributed to the high consistency observed.

Furthermore, although Friberg et al. [19] included a smaller sample size than our study, the lower accuracy in older investigations may also be attributed to the lower measurement precision of radiographic equipment available in the late 1980s. More recently, Gross et al. [42] reported high intra-examiner reliability (ICC = 0.84) for the Blocks Method, which aligns more closely with our results. However, given the pilot nature of this study, the Modified ICPBL test has not been identified as a ‘definitive replacement’ for telemetry; instead, it is proposed as a highly effective screening tool [2].

A distinction must be made between the diagnostic detection threshold of the Modified ICPBL test and what constitutes a clinically meaningful discrepancy. Our findings demonstrate that the modified ICPBL test is highly accurate in detecting anatomical differences as small as 1.88 mm relative to the 3 mm gold-standard cut-off. However, the presence of a detectable anatomical discrepancy does not inherently equate to clinical impairment. While a 3 mm difference is technically diagnosable and serves as a robust benchmark for validating new clinical tools, its clinical significance—specifically the point at which it triggers symptoms such as low back pain or gait deviations—remains subject to individual compensatory capacity and activity levels [7,8]. In this regard, Resende et al. [1] demonstrated that even mild leg length discrepancies induce immediate biomechanical strategies during gait to maintain pelvis and trunk stability, supporting the relevance of detecting subtle anatomical deviations. Therefore, the modified ICPBL test should be viewed as a high-precision screening tool for identifying the existence of DA, whereas the decision to implement treatment should be based on the clinical correlation of these findings with the patient’s symptoms.

### 4.2. Plantar Pressures

The plantar pressure values demonstrated sufficient reliability to support a valid screening of DA, though performance metrics were lower than the Modified ICPBL. In the literature, platforms have shown reliable validity [32], yet most evidence originates from simulated discrepancies rather than true DA subjects [21,43]. Most investigations using blocks or lifts of 1–2 cm [22] produce discrepancies larger than those observed in true clinical cases, potentially introducing bias.

Current evidence suggests that substantial kinematic adaptations emerge predominantly when discrepancies reach 2 cm or more [22]. In contrast, our study identified measurable alterations at 2.83 kg/cm^2^, consistent with reports [40,44] of subtle shifts in the center of pressure. The heterogeneity in redistribution reported in the literature—where some studies find increased excursion under the longer limb and others under the shorter [15,34,35]—likely reflects individualized neuromuscular control based on body mass and age. This is consistent with evidence showing that the body implements specific kinematic compensations at the ankle and hip to functionally equalize even slight anatomical differences [45]. From a biomechanical perspective, the link between DA and plantar pressure asymmetry resides in the shift in the body’s center of mass (CoM) [40,41]. Theoretically, an anatomical discrepancy creates a frontal plane pelvic tilt that displaces the CoM toward the shorter limb as a strategy to minimize energy expenditure during static stance [7,12]. This shift increases the vertical ground reaction forces on the shorter side, which our study captured as a significant pressure increase (cutoff of 2.83 kg/cm^2^). When comparing our results with orthopedic imaging literature, our findings align with radiographic studies showing that even sub-centimeter discrepancies (3–5 mm) alter the load-bearing axis of the lower limbs. While orthopedic surgery often considers 10 mm as the threshold for intervention, our podobarometric data suggests that the kinetic chain begins to redistribute loads at much lower anatomical thresholds (1.88–3 mm), supporting the use of functional assessments alongside traditional imaging.

The moderate sensitivity (69.2%) observed aligns with the inherent variability of true DA subjects [15,16], whereas simulated models tend to exaggerate changes [35,42]. Regarding predictive values (PPV and NPV), and consistent with Heath et al. [46], reliability appears to be influenced by prevalence. Overall, these findings support the cautious but meaningful application of plantar pressure platforms in early DA detection.

### 4.3. Limitations

The present study has several limitations that should be acknowledged to ensure a balanced interpretation of the findings.

First, the relatively small sample size (n = 30) defines this investigation as a pilot study. While the high intra-class correlation coefficients (ICC) and narrow confidence intervals (CI) suggest strong internal consistency, the limited number of participants may restrict the statistical power and the generalizability of the results to the broader population. As noted in high-impact methodological reviews, when the complexity of the analysis (e.g., ROC curves and Bland–Altman plots) is high relative to the number of positive cases, there is an inherent risk of overfitting [39]. In such cases, the model may capture random noise or specific data alignments rather than a true universal biological signal. Consequently, the results of “perfect accuracy” for the Modified ICPBL test should be regarded as preliminary and indicative of high potential rather than an infallible clinical reality.

Second, the assessment was limited to static conditions. Both the Modified ICPBL test and the plantar pressure analysis were performed in a standing position. While these provided a valid screening for DA, they do not account for the compensatory mechanisms and kinematic adaptations that occur during the dynamics of gait. Future research incorporating dynamic pressure testing and 3D motion analysis would be essential to verify these results under more functional conditions.

Third, inter-examiner variability was not evaluated. All clinical measurements were conducted by a single examiner with over 15 years of experience, which likely enhanced the consistency of the results. However, the reproducibility of the Modified ICPBL test across different clinicians with varying levels of training remains untested. Future multi-center studies should include multiple raters to confirm the inter-examiner reliability and external validity of the method.

Fourth, potential selection bias must be acknowledged, as participants were recruited from a specific clinical setting using convenience sampling. In accordance with the STARD 2015 guidelines [39], we recognize that this recruitment strategy may not fully represent the broad phenotypic spectrum of patients encountered in general orthopedic or podiatric practice. Although this risk was mitigated through a rigorous inclusion protocol and a highly homogeneous sample regarding age and BMI to control for confounding biomechanical variables, the findings may not be generalized to individuals with complex multi-planar pathologies, previous surgical interventions, or severe structural asymmetries. Furthermore, the cohort was dichotomized using a 3 mm diagnostic threshold for DA, a value firmly established in the literature [11]; however, this binary classification may overlook the nuances of progressive compensatory mechanisms. Therefore, subsequent research should prioritize stratified analyses based on the severity of the discrepancy (e.g., mild, moderate, and severe subgroups) to determine whether the diagnostic accuracy and clinical sensitivity of the Modified ICPBL test fluctuate across different magnitudes of anatomical deviation.

In summary, while these findings provide a robust framework for the clinical screening of DA, they represent an initial stage of validation. Larger, diverse, and multi-center cohorts are required to confirm the reliability and clinical applicability of these non-radiological diagnostic tools.

## 5. Conclusions

According to our results, the Modified ICPBL and plantar pressure tests demonstrated high diagnostic accuracy within the studied sample for diagnosing DA. These may serve as complementary screening methods to the gold standard TE, potentially reducing subjects’ exposure to X-ray radiation in cases of suspected lower limb asymmetry. The cut-off values identified were 2.83 kg/cm^2^ for the plantar pressure test and 1.88 mm for the Modified ICPBL. While the modified ICPBL showed the highest performance metrics in this pilot cohort, these findings must be interpreted with caution due to the limited sample size and the specific clinical setting. Future studies should include larger, multi-center cohorts and multiple examiners to validate these preliminary results, evaluate plantar pressure measurements under dynamic conditions, and include patients with varying severities of limb length discrepancy to better characterize the diagnostic sensitivity of these tools across the full clinical spectrum of DA.

## Figures and Tables

**Table 1:** Descriptive table presenting the sample population’s sociodemographic data and specific clinical characteristics.

Variable	Total Sample (N = 30)	DA Group (n = 13)	Non-DA Group (n = 17)	*p*-Value
Age (years)	51.60 ± 15.41	52.77 ± 14.45	50.71 ± 16.50	718
Height (cm)	168.43 ± 9.42	168.77 ± 9.16	168.18 ± 9.89	867
Weight (kg)	71.34 ± 11.07	73.10 ± 8.73	70.00 ± 12.68	435
TE Gold Standard (mm)	3.73 ± 4.45	8.09 ± 3.24	0.41 ± 0.96	<0.001 *
Modified ICPBL (mm)	2.19 ± 2.40	4.38 ± 2.10	0.51 ± 0.53	<0.001 *
Plantar Pressures (kg/cm^2^)	3.53 ± 2.87	5.17 ± 3.28	2.28 ± 1.77	0.024 *

**Abbreviations:** N = number of subjects; *p*-value = probability that the given value is statistically significant; *p* < 0.05 (with a 95% confidence interval); ICPBL = palpation of iliac crests and correction with blocks; DA = anatomic discrepancy; TE = telemetry; mm = millimeters; Kg/cm^2^ = kilograms per square centimeter. * = statistically significant.

**Table 2:** Intraclass correlation coefficient between the techniques and intra-examiner measurements and optimal cut-off values and the ability of each variable to predict the Discrepancy.

Technique Variable	ICC (3,1) (95% CI)	ICC (3,1) Intra (95% CI)	Optimal Cut-Off Value	Area Under the ROC Curve (95% CI) (Lb–Ub)	*p*-Value	Sensitivity % (95% CI) (Lb–Ub)	Specificity % (95% CI) (Lb–Ub)
	(Lb–Ub)	(Lb–Ub)					
PLANTAR PRESSURES (kg/cm^2^)	0.741 (0.456–0.877)	0.911 (0.844–0.953)	2.83	0.783 (0.603–0.962)	<0.05 *	69.2 (38.6–90.9)	82.4 (56.6–96.2)
MODIFIED ICPBL (mm)	0.877 (0.742–0.942)	0.977 (0.959–0.988)	1.88	1.000 (0.912–1.000)	<0.001 **	100 (75.3–100)	100 (80.5–100)

**Abbreviations:** CI = Confidence Interval. 95% CI for sensitivity and specificity were calculated using the Clopper–Pearson exact method. Statistical power was calculated a posteriori based on an expected area under curve of 0.80, yielding a power of 84.1% (α = 0.05); *p*-value * = probability that the given value is statistically significant; *p* < 0.05 (with a 95% confidence interval); ICC (3,1) = Intraclass Correlation Coefficient using a two-way mixed-effects model, absolute agreement, and single-measures. 95% CI: 95% Confidence Interval. Interpretation of ICC values followed Koo and Li guidelines [36]; Lb = lower bound; Ub = upper bound; ICC-intra = Intraclass correlation coefficient intra-examiners; *p*-value ** = probability that the given value is strongly statistically significant; *p* < 0.001 (with a 95% confidence interval); Modified ICPBL = palpation of iliac crests and correction with blocks; mm = millimeters; Kg/cm^2^ = kilograms per square centimeter.

**Table 3:** Error estimation table and standard error for both Plantar Pressures and Modified ICPBL test.

Test	Actual Condition (TE)	Predicted: NO	Predicted: YES	Accuracy/Metric
Plantar Pressures	Negative (Non-DA)	14	3	Specificity: 82.4%
(Cut-off: 2.83 kg/cm^2^)	Positive (DA)	4	9	Sensitivity: 69.2%
AUC: 0.783	Total Accuracy			76.7%
---	---	---	---	---
Modified ICPBL	Negative (Non-DA)	17	0	Specificity: 100%
(Cut-off: 1.88 mm)	Positive (DA)	0	13	Sensitivity: 100%
AUC: 1.000	Total Accuracy			100%

**Abbreviations:** AUC: area under the curve.

**Table 4:** Positive and Negative Predictive Values of Plantar Pressures and Modified ICPBL Tests at Different Prevalences of Anatomical Discrepancy.

Test	Prevalence	PPV (%)	NPV (%)
Plantar pressures	35.5%	67.9	82.9
Plantar pressures	50%	79.7	72.8
Plantar pressures	80%	94.0	40.1
Modified ICPBL	35.5%	100	100
Modified ICPBL	50%	100	100
Modified ICPBL	80%	100	100

**Abbreviations:** PPV = positive predictive values; NPV = negative predictive values.

## Data Availability

The raw data supporting the conclusions of this article will be made available by the authors on request.
